# Evolutionarily Conserved Repulsive Guidance Role of Slit in the Silkworm *Bombyx mori*

**DOI:** 10.1371/journal.pone.0109377

**Published:** 2014-10-06

**Authors:** Qi Yu, Xiao-Tong Li, Chun Liu, Wei-Zheng Cui, Zhi-Mei Mu, Xiao Zhao, Qing-Xin Liu

**Affiliations:** 1 Laboratory of Developmental Genetics, Shandong Agricultural University, Tai'an, Shandong, China; 2 State Key Laboratory of Silkworm Genome Biology, Southwest University, Chongqing, China; Trinity College Dublin, Ireland

## Abstract

Axon guidance molecule Slit is critical for the axon repulsion in neural tissues, which is evolutionarily conserved from planarians to humans. However, the function of Slit in the silkworm *Bombyx mori* was unknown. Here we showed that the structure of *Bombyx mori* Slit (BmSlit) was different from that in most other species in its C-terminal sequence. BmSlit was localized in the midline glial cell, the neuropil, the tendon cell, the muscle and the silk gland and colocalized with BmRobo1 in the neuropil, the muscle and the silk gland. Knock-down of *Bmslit* by RNA interference (RNAi) resulted in abnormal development of axons and muscles. Our results suggest that BmSlit has a repulsive role in axon guidance and muscle migration. Moreover, the localization of BmSlit in the silk gland argues for its important function in the development of the silk gland.

## Introduction

The Slit protein plays an important role in a variety of physiological and pathological processes, such as nervous system development, heart morphogenesis, muscle migration and tumor metastasis [1]–[6]. The *slit* gene was first identified in *Drosophila* and its homologues have been found in many species. There is a single *slit* in invertebrates. Three *slit* genes, *slit1*, *slit2*, and *slit3* are found in tetrapods and there are four *slit* genes in teleosts [7]–[11]. It is generally considered that during animal evolution there is a global trend towards increases in gene size, complexity, and diversity [12]. Therefore, it is necessary to understand to what extent the evolutionary diversification of the *slit* gene contributes to the increase in the complexity of animals.

In insects, much of our knowledge about functions of the *slit* gene has come from studies in the dipteran *Drosophila melanogaster* [5], [13], [14]. Recently, the investigation of *slit*-mediated axon guidance has been extended to the coleopteran *Tribolium castaneum* [15]. The silkworm *Bombyx mori* has served as a lepidopteran model animal with many experimental advantages, such as large body size, short lifecycle, ease of rearing and rich genetic resources [16]. In addition, the completion of silkworm genome sequencing facilitates studies on molecular biology [17]. Separated by more than 240 million years from *D. melanogaster*, *B*. *mori* provides an important window on certain evolutionary changes of the lepidoptera relative to the diptera [16]. However, as a crucial guidance molecule for diverse cell types, the *slit* gene in the silkworm has not been described.

Here we report the characterization of *Bmslit*, a *slit* orthologue from *B. mori*. Multiple sequence alignment and domain analysis indicate that BmSlit has a relatively conserved structure but with a different form of C-terminal sequence. BmSlit localizes to the midline glial cell, the neuropil, the tendon cell, the muscle, and the silk gland and plays an important role in axon guidance and muscle migration in *B. mori*.

## Materials and Methods

### Experimental Animals

The silkworm strains Zhg×Chun54, 9202×Lu7 and Dazao were used in this study. Eggs were incubated at 25°C. Larvae were reared on fresh mulberry leaves or artificial diet at 25°C.

Identification and Cloning of *Bmslit*

To identify the *slit* orthologue in the silkworm, the fruit fly Slit protein sequence was used as the query sequence to perform BLAST search against the silkworm genome database (http://silkworm.genomics.org.cn/). Total RNA was extracted from the brain of day 3 fifth instar larvae. The first-stranded cDNA was synthesized using reverse transcriptase AMV (Roche) and an initial fragment of *Bmslit* was amplified by PCR using Primer 1F and Primer 1R (Table S1). Rapid amplification of cDNA ends (RACE) was performed using primers (5′-RACE: Primer 2-1 and Primer 2-2; 3′-RACE: Primer 3-1 and Primer 3-2) (Table S1) according to the manufacturer's instructions of the SMART PCR cDNA Amplification kit (Clontech).

### Sequence Analysis

Protein sequence alignment was performed by DNASIS MAX Version 3.0 (MiraiBio, San Francisco, CA). Domain architectures for BmSlit were determined by SMART [18]. To investigate the evolutionary relationships between Slit of *B. mori* and other organisms, the neighbor joining (NJ) tree with Poisson model was constructed using MEGA5 [19].

### Generation of Anti-BmSlit Antibody

The nucleotide sequence encoding 107 amino acids at the C-termini of BmSlit was amplified by PCR using Primer 4-F and Primer 4-R (Table S1). The PCR product was cloned into the expression vector pET28a and transformed into *Escherichia coli* BL21 (DE3) cells. The fusion protein was purified by HisTrap HP column (GE Healthcare) and used to generate polyclonal antibodies in mice (AbMax Biotechnology, Beijing, China).

### In Situ Hybridization

In situ hybridization was carried out as previously described [20]. A 570 bp DNA fragment of *Bmslit*, residing in the fifth EGF domain to the LamG domain, was amplified by PCR using Primer 5-F and Primer 5-R (Table S1) and cloned into pGEM-T Easy Vector (Promega). *Bmslit* probe was generated by digesting the recombinant plasmid with *Nco*I and transcribing with DIG RNA Labeling Kit (Roche).

### Western Blot Analyses

Western blot was carried out as previously described [21]. The primary antibody was anti-BmSlit antibody (1∶100) and the secondary antibody was horseradish peroxidase-conjugated goat anti-mouse IgG antibody (1∶20000, Jackson ImmunoResearch).

### Immunofluorescence Staining

Antibody staining was carried out as previously described [22]. Whole embryos or silk glands were dissected in PBS and fixed in 4% formaldehyde in PEM buffer (PEM: 100 mM Pipes-KOH at pH 7.0, 2 mM EGTA, 1 mM MgSO4) for 40 minutes on ice, then permeabilized for 15 minutes at room temperature in PBS containing 0.5% NP40, and blocked for 2 hours in PBS containing 0.1% BSA and 5% goat serum. The samples were stained with the primary antibodies for 2 hours, followed by incubation with secondary antibodies for 1.5 hours at room temperature. The primary antibodies we used were as follows: anti-BmSlit antibody (1∶100), anti-BmRobo1 antibody (1∶100, developed by our lab) and mouse monoclonal antibody 22C10 (1∶20, Developmental Studies Hybridoma Bank). The secondary antibodies Alexa 488-conjugated goat anti-rabbit IgG (Jackson ImmunoResearch) and Cy3-conjugated donkey anti-mouse IgG (Jackson ImmunoResearch) were used at a dilution of 1∶500. The anti-HRP-FITC antibody (1∶200, Jackson Immunoresearch) was also used.

### RNA Interference

The same sequence with the in situ hybridization probe was used for the *Bmslit* RNA interference. Double-stranded RNA (dsRNA) of *Bmslit* and enhanced green fluorescent protein (EGFP) gene was synthesized in vitro by RiboMAX Large Scale RNA Production Systems (Promega). About 3 nl of 1 µg/µl *Bmslit* dsRNA solution was injected into silkworm embryos, which were collected within 3 hours of oviposition. The same amount of *egfp* dsRNA was used as a control.

### Microscopy and Image Treatment

Images were acquired by the laser scanning confocal microscope (Leica SD AF) and fluorescence microscope (Olympus BX53) and treated with Adobe Photoshop CS6 image programs. For confocal microscopy, the step size of stacks was 1 µm.

## Results

### Isolation and Sequence Analysis of *Bmslit*

**Figure 1:**
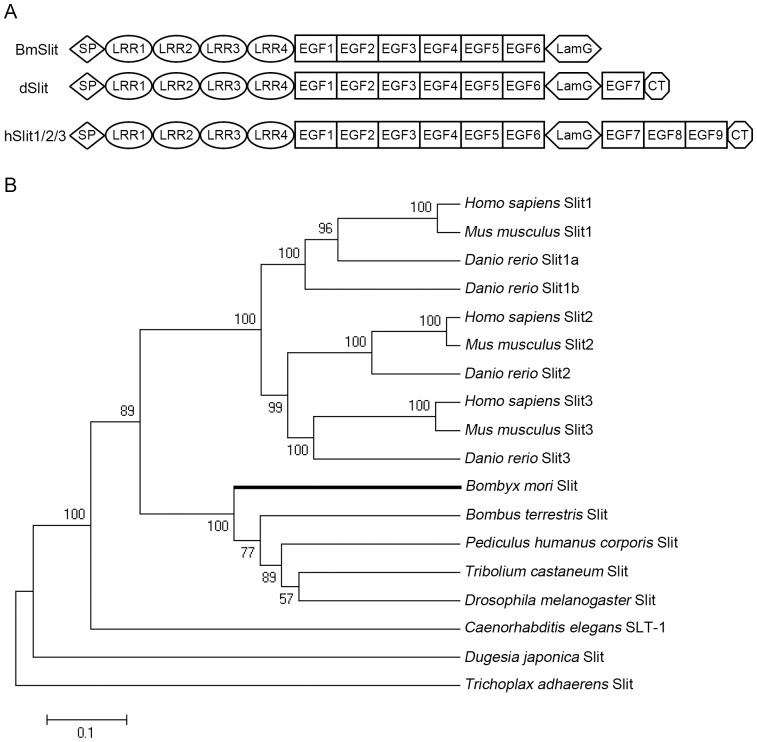
Sequence analysis of BmSlit. (A) Protein structure comparison of *Bombyx mori* Slit (BmSlit), *Drosophila melanogaster* Slit (dSlit) and *Homo sapiens* Slits (hSlit1, hSlit2, hSlit3). (B) Phylogenetic tree of Slit. Numbers next to the branches indicate bootstrap values with 1000 replicates. The scale bar represents a distance of 0.1 amino acid substitutions per site.

*Bmslit* was located on chromosome 24 of *B*. *mori* according to the silkworm genome database. The full length cDNA of *Bmslit* was isolated from the brain of silkworm, which has been submitted to GenBank (accession number KF739412). The ORF (open reading frame) sequence of *Bmslit* consisted of 4002 bp, encoding 1333 amino acid residues. The predicted BmSlit protein contained four leucine-rich repeats (LRRs), six epidermal growth factor-like (EGF) repeats and a laminin G (LamG) domain, lacking the seventh EGF domain and the C-terminal cysteine-rich (CT) domain comparing with its homologue in *Drosophila* (Figure 1A and Figure S1). A phylogenetic tree was constructed using the Slit protein sequences from *B*. *mori* and other species (Figure 1B). The phylogenetic analysis indicates that the *Bmslit* gene is orthologous to the *slit* genes of other species.

### Expression Pattern of *Bmslit*

**Figure 2:**
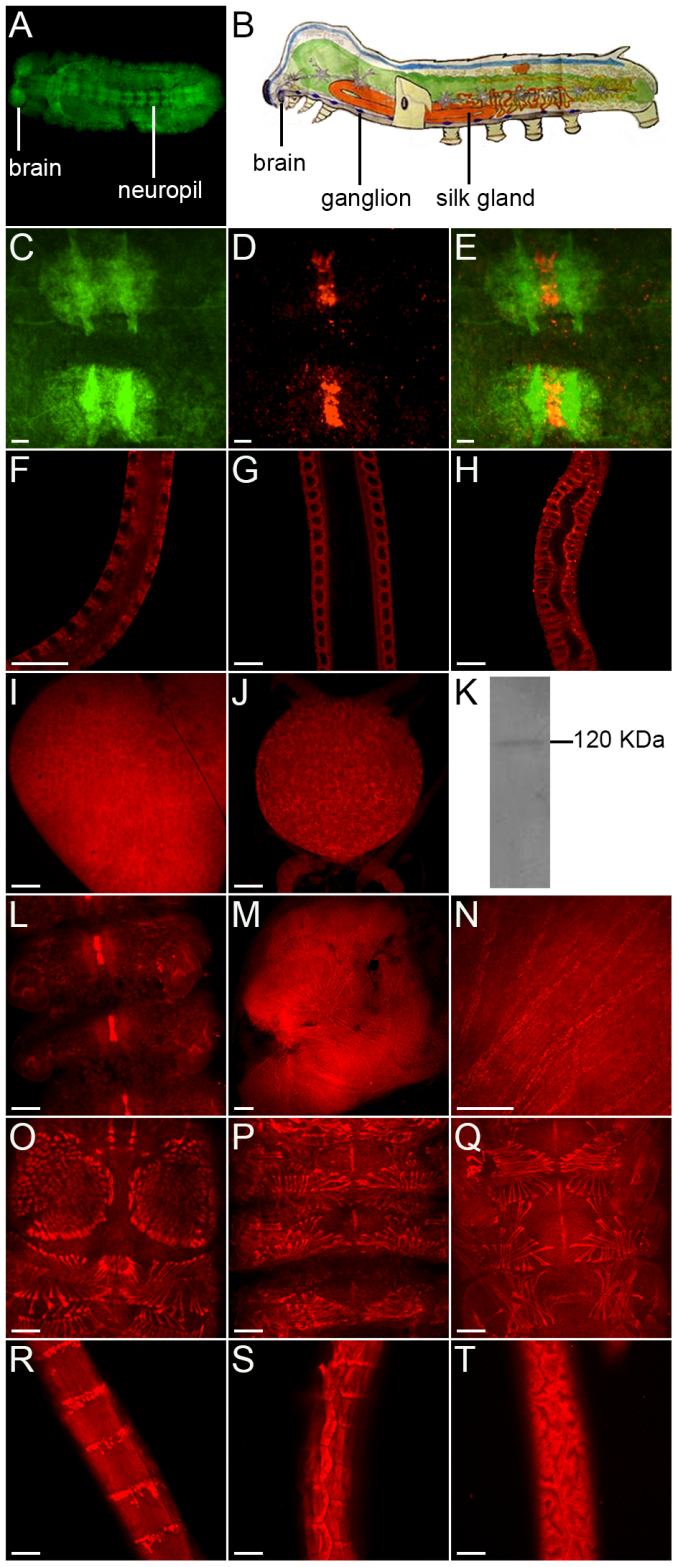
Expression pattern of *Bmslit*. (A) The central nervous system of a stage 20 silkworm embryo stained with the anti-HRP-FITC antibody. (B) A schematic of the brain, the ganglion, and the silk gland of the silkworm larva. (C–D) *Bmslit* mRNA was expressed in the midline glial cell of a stage 22 silkworm embryo (D) and the central nervous system was revealed by anti-HRP-FITC antibody (C). (E) The merged image of (C) and (D). (F–J) *Bmslit* mRNA was expressed in the anterior silk gland (F), the middle silk gland (G), the posterior silk gland (H), the brain (I) and the ganglion (J) of day 2 first instar larvae. (K) Western blot analysis of BmSlit in the newly-hatched silkworm. (L–N) Localization of BmSlit in the midline glial cell and the neuropil of embryos (L), the brain (M) and the ganglion of larvae (N). The stage 22 embryo and day 3 fifth instar larvae were stained by anti-BmSlit antibody. (O–Q) Localization of BmSlit in the tendon cell and muscle of anterior segments (O), intermediate segments (P), and posterior segments (Q). The stage 23 embryo was stained by anti-BmSlit antibody. (R–T) Localization of BmSlit in the anterior silk gland (R), the middle silk gland (S), and the posterior silk gland (T). The silk gland of day 3 fifth instar larvae was stained with anti-BmSlit antibody. Scale bars represent 200 µm (C–J), 400 µm (L, N–T) and 50 µm (M).

The structure of the central nervous system of silkworm embryo was stained by anti-HRP-FITC antibody (Figure 2A) and structures of the brain, the ganglion and the silk gland of silkworm larvae were shown in Figure 2B. To analyze the expression pattern of *Bmslit* in these tissues, we performed in situ hybridization. *Bmslit* mRNA was expressed at the midline glial cell of the stage 22 embryo (Figure 2C–E), and the silk gland (Figure 2F–H), the brain (Figure 2I) and the ganglion (Figure 2J) of day 2 first instar larvae.

The expression of BmSlit in the newly-hatched silkworm was performed by western blotting. As shown in figure 2K, a single band of 120 KDa was detected. To determine the localization of BmSilt, we performed tissue staining using the anti-BmSlit antibody. BmSlit was detected in the midline glial cell and the neuropil (Figure 2L) of the stage 22 embryo, the brain (Figure 2M) and the ganglion (Figure 2N) of day 3 fifth instar larvae, the tendon cell and muscle of the stage 23 embryo (Figure 2O–Q), and the silk gland of day 3 fifth instar larvae (Figure 2R–T). These results suggest that BmSlit is involved in the development of diverse tissues.

### Colocalization of BmSlit and BmRobo1

**Figure 3:**
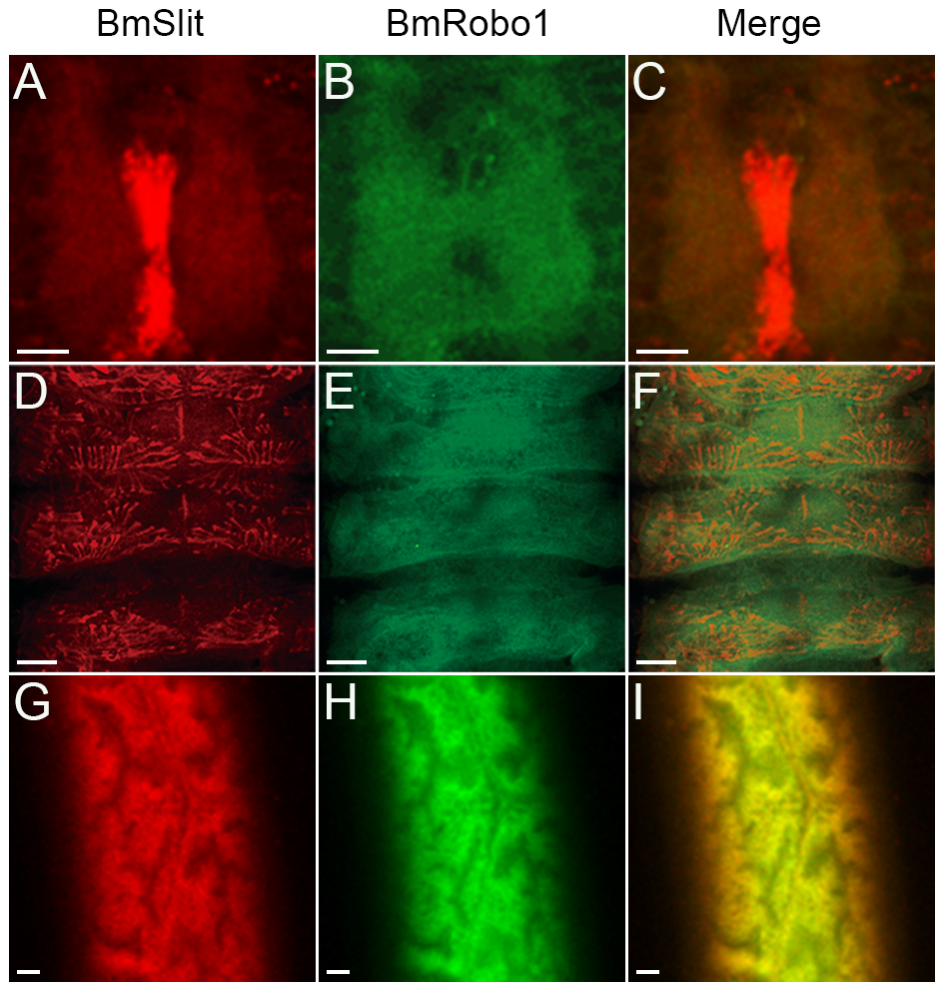
Colocalization of BmSlit and BmRobo1. (A–C) BmSlit (A) and BmRobo1 (B) were colocalized in the neuropil. (D–F) BmSlit (D) and BmRobo1 (E) were colocalized in the muscle. (G–I) BmSlit (G) and BmRobo1 (H) were colocalized in the silk gland. (C, F and I) Merged images of (A) and (B), (D) and (E), (G) and (H), respectively. Embryos of stage 22 and 23 and the silk gland of day 3 fifth instar larvae were stained by anti-BmSlit and anti-BmRobo1 antibodies. Scale bars represent 100 µm (A–C, G–I) and 400 µm (D–F).

It has been established that Slit signals through the Robo receptor [1], [13]. To test the colocalization of BmSlit and BmRobo1, we performed a double-staining experiment. The results showed that BmSlit and BmRobo1 were colocalized in the neuropil (Figure 3A–C), the muscle (Figure 3D–F) and the silk gland (Figure 3G–I). The colocalization of BmSlit and BmRobo1 suggests their roles in the development of the neuron, the muscle and the silk gland.

BmSlit-mediated Repulsion of Axon Guidance and Muscle Migration

**Figure 4:**
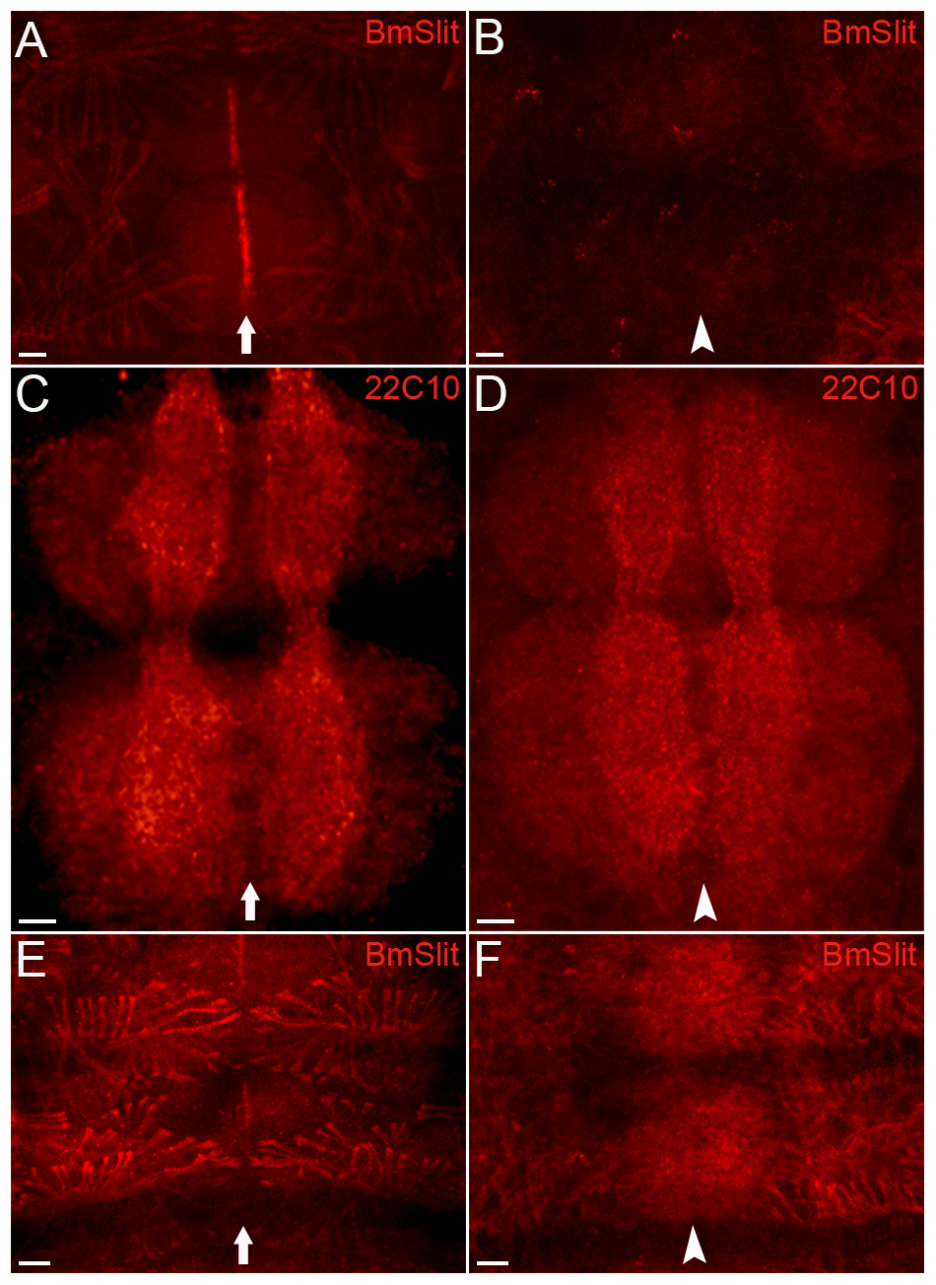
Knock-down phenotypes of *Bmslit*. (A) The embryos injected with *egfp* dsRNA were stained with anti-BmSlit antibody. The expression of BmSlit in midline glial cells was not affected (arrow). (B) The embryos injected with *Bmslit* dsRNA were stained with anti-BmSlit antibody. The expression of BmSlit in midline glial cells was significantly reduced (arrowhead). (C) In embryos injected with *egfp* dsRNA (as a control for the RNAi method) and stained with 22C10 antibody, the axons were visible on each side of the midline (arrow) (100%, n = 78). (D) The axon phenotype of embryos injected with *Bmslit* dsRNA (82.5%, n = 80). The axons were closer to the midline than in the control animal (arrowhead). (E) In embryos injected with *egfp* dsRNA and stained with anti-BmSlit antibody, the muscles were visible on each side of the midline (arrow) (100%, n = 76). (F) The muscle phenotype of embryos injected with *Bmslit* dsRNA (80%, n = 75). Some muscles crossed the midline (arrowhead). Scale bars represent 100 µm.

To investigate the function of BmSlit, we used RNAi to silence expression of *Bmslit*. The expression of BmSlit in midline glial cells of embryos injected with *egfp* dsRNA was not affected (Figure 4A). However, the expression of BmSlit in midline glial cells of embryos injected with *Bmslit* dsRNA was significantly reduced (Figure 4B). In the control embryo, the neuropil was situated at a particular distance from the midline on either side (Figure 4C). In the *Bmslit* RNAi embryo, the bilateral axons appeared to be closer to the midline compared with the control (82.5%, n = 80) (Figure 4D). The mean (SD) intervals between the left and right axon bundle were 550 (7.5) µm in the *Bmslit* RNAi embryos and 630 (9) µm in the control embryos, with a significant difference (P<0.01). The muscles were arranged in a symmetrical pattern with some distance from the midline in the control embryo (Figure 4E). In the *Bmslit* RNAi embryo, the arrangement of muscles became irregular and some muscles crossed the midline (80%, n = 75) (Figure 4F). These results suggest that BmSlit is a repulsive cue in axon guidance and muscle migration of the silkworm.

## Discussion

Slit is an evolutionarily conserved multifunctional protein and its guidance role has been studied in many organisms, such as planarians, nematodes, flies, and vertebrates [2], [5], [8], [9]. In this study, we identified and characterized an orthologue of the *slit* gene from the silkworm *B*. *mori*.

It has been known that the interaction between Slit and its receptor Robo is mediated through the second LRR domain of Slit and the first Ig domain of Robo [23]. Comparing with the *Drosophila* counterpart, BmSlit lacked the seventh EGF domain and the CT domain, but it was similar to the protein structure of *Dugesia japonica* Slit. Multiple sequence alignment showed that the LRR2 domain of BmSlit is clearly conserved. Therefore, although BmSlit lacked the partial C-terminal sequence, the interaction between BmSlit and its receptor may not be affected. We also found the colocalization of BmSlit and BmRobo1, which further supported the ligand-receptor relationship between BmSlit and BmRobo1.

As a multifunctional molecule, the Slit protein controls the development of diverse tissues [24]. We showed that BmSlit was localized in the midline glial cell, the neuropil, the tendon cell, the muscle and the silk gland. RNAi-mediated knockdown of *Bmslit* produced abnormal arrangements of axons and muscles. These results indicate that BmSlit contributes to the development of axons and muscles. In addition, surprisingly, we found that BmSlit colocalized with BmRobo1 in the silk gland. The silk gland is derived from the labial segment and then migrates dorsally and posteriorly [25]. So far, the molecular mechanism underlying the migration of silk gland is unclear. However, our finding raises an intriguing possibility that BmSlit is involved in the migration of the silk gland.

## Supporting Information

Figure S1 **Sequence alignment of** ***Bombyx mori*** **Slit with** ***Drosophila melanogaster*** **Slit (NP_476727.1) and** ***Homo sapiens*** **Slits (Slit1, NP_003052.2; Slit2, NP_004778.1; Slit3, NP_001258875.1).** Species are abbreviated as: Bm, *Bombyx mori*; d, *Drosophila melanogaster*; h, *Homo sapiens*.
(TIF)

Table S1 **Primer sequences used in this study.**
(DOC)
