# Supplementary figures and images for: Evolutionarily Conserved Repulsive Guidance Role of Slit in the Silkworm Bombyx mori
Source: PLoS One. 2014 Oct 6;9(10):e109377. doi: 10.1371/journal.pone.0109377 (PMC4186835; doi:10.1371/journal.pone.0109377)

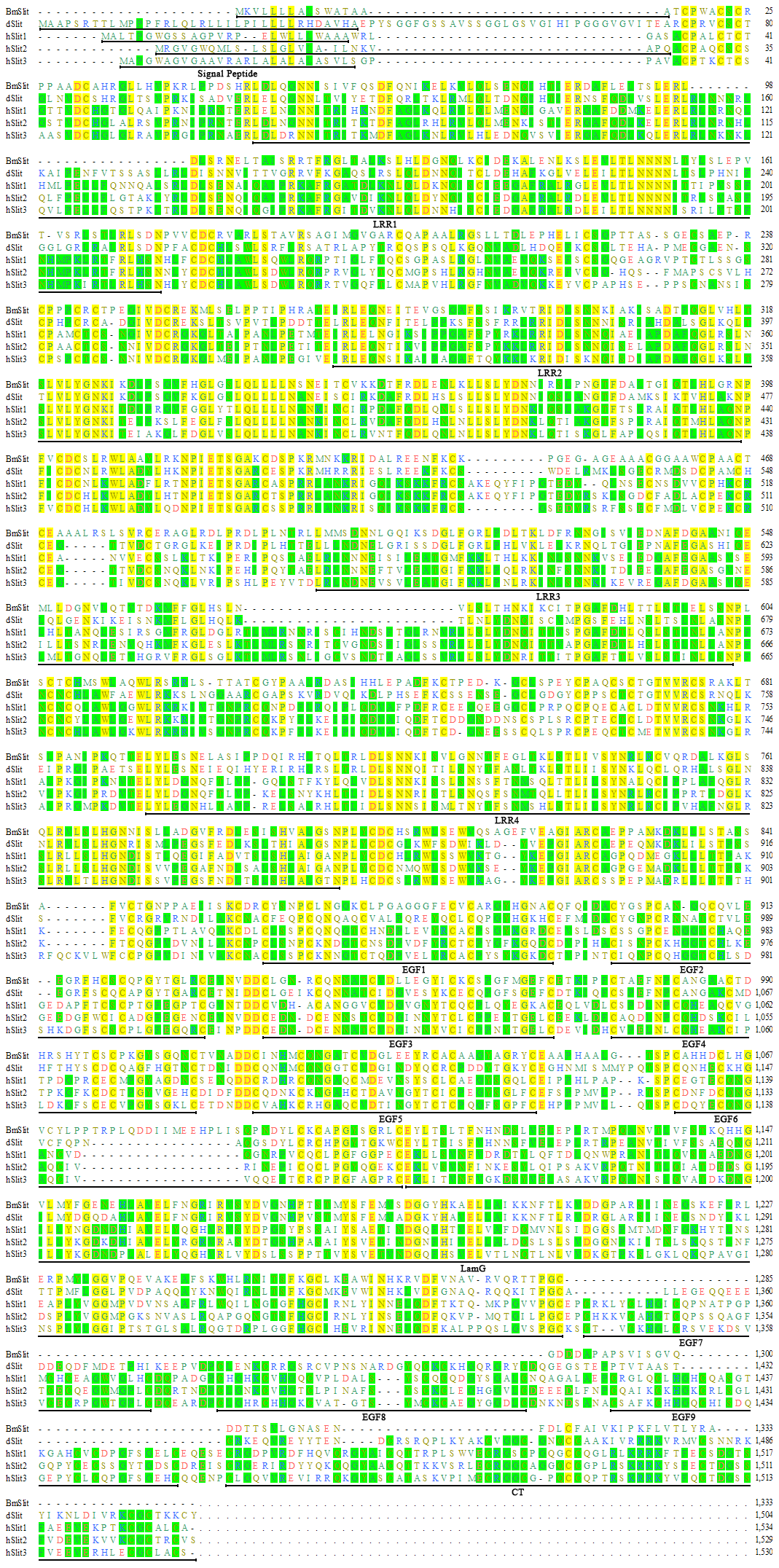

Supplement: Figure S1 — Sequence alignment of Bombyx mori Slit with Drosophila melanogaster Slit (NP_476727.1) and Homo sapiens Slits (Slit1, NP_003052.2; Slit2, NP_004778.1; Slit3, NP_001258875.1). Species are abbreviated as: Bm, Bombyx mori; d, Drosophila melanogaster; h, Homo sapiens. (TIF) [file pone.0109377.s001.tif]
