# Supplementary material for: Evolutionarily Conserved Repulsive Guidance Role of Slit in the Silkworm Bombyx mori
Source: PLoS One. 2014 Oct 6;9(10):e109377. doi: 10.1371/journal.pone.0109377 (PMC4186835; doi:10.1371/journal.pone.0109377)
Supplement: Table S1 — Primer sequences used in this study. (DOC) [file pone.0109377.s002.doc]

Table S1. Primer sequences used in this study.

| Primer name | Primer sequence (5’-3’) |
| --- | --- |
| Primer 1-F | GTCTTGGAAGAGGGCAGGTT |
| Primer 1-R | GCCTTCGTTGATGATGGATC |
| Primer 2-1 | CGTGCAGAACGGTATCTTCGTCTCGC |
| Primer 2-2 | CAGTGGAACCTGCCCTCTTCCAAGAC |
| Primer 3-1 | TGCTCATGTACTTCGGTGAGAACGAGC |
| Primer 3-2 | GCCAGATCCATCATCAACGAAGGCAGC |
| Primer 4-F | CAAGCTTGGATCCCTAGAGAGACCGATGTACCT |
| Primer 4-R | CAAGCTTAAGCTTTCACGCTCGATATAGTGTGAC |
| Primer 5-F | GAGGAGTATCGGTGCGCAT |
| Primer 5-R | GCCTTCGTTGATGATGGAT |
